# Extraction of Step-Feature Lines in Open-Pit Mines Based on UAV Point-Cloud Data

**DOI:** 10.3390/s22155706

**Published:** 2022-07-30

**Authors:** Yachun Mao, Hui Wang, Wang Cao, Yuwen Fu, Yanhua Fu, Liming He, Nisha Bao

**Affiliations:** 1 School of Resources & Civil Engineering, Northeastern University, Shenyang 110819, China; maoyachun@mail.neu.edu.cn (Y.M.); dbdxcw@163.com (W.C.); fuyuwen_neu@163.com (Y.F.); heliming@mail.neu.edu.cn (L.H.); baonisha@mail.neu.edu.cn (N.B.); 2 School of Architecture, Northeastern University, Shenyang 110819, China; fuyanhua@mail.neu.edu.cn

**Keywords:** three-dimensional point cloud, open-pit mines, step-feature lines, gradient edge detection

## Abstract

Step-feature lines are one of the important geometrical elements for drawing the status quo maps of open-pit mines, and the efficient and accurate automatic extraction and updating of step-feature lines is of great significance for open-pit-mine stripping planning and analysis. In this study, an automatic extraction method of step-feature lines in an open-pit mine based on unmanned-aerial-vehicle (UAV) point-cloud data is proposed. The method is mainly used to solve the key problems, such as low accuracy, local-feature-line loss, and the discontinuity of the step-feature-line extraction method. The method first performs the regular raster resampling of the open-pit-mine cloud based on the MLS algorithm, then extracts the step-feature point set by detecting the elevation-gradient change in the resampled point cloud, further traces the step-feature control nodes by the seed-growth tracking algorithm, and finally generates smooth step-feature lines by fitting the space curve to the step-feature control nodes. The results show that the method effectively improves the accuracy of step-feature-line extraction and solves the problems of local-feature-line loss and discontinuity.

## 1. Introduction

Intelligent mining is an important theme in current mining-resource development. Realizing the automation and intelligence of mining, and ensuring efficient and intelligent mining, are the main development trends of smart mines. The open-pit-mining status map is an important basis for the spatial-information description, production planning, and mining-acceptance measurement of the open-pit mine, and it is also an important premise for ensuring efficient and intelligent mining. The current open-pit mine describes the spatial information of the open-pit mine and the mining status of the open pit through the step-feature line. In recent years, UAV 3D point-cloud-data acquisition and processing technology [1,2,3] has been widely used in the field of 3D modeling, and in the spatial analysis of open-pit mines after continuous and rapid development [4,5,6,7]. Although UAV point-cloud technology has made great contributions to mine automation, step-feature-line drawing is still mainly realized by manual visual interpretation. The surveyor first needs to obtain the mine point-cloud data and generate the digital mine model by means of drones, it then determines the step-feature points by means of visual interpretation, and it finally outlines the step-feature line by hand. This method is cumbersome, poor in measurement accuracy, and low in automation, which makes it difficult to meet the needs of the fast-developing modern society for mining automation and intelligence, which is contrary to the main development trend of intelligent mining. Therefore, how to extract the step-feature lines quickly and accurately based on the point clouds of open-pit mines becomes a critical problem to be solved in mine automation applications.

The step-feature lines of a surface open-pit mine can be described by using the 3D edge features of the steps. The extraction of 3D edge features is usually achieved by retaining feature data, such as contour lines, concave and convex fold lines, and transition smooth lines that reflect the geometry of the feature. The 3D edge-feature-extraction methods can be summarized as follows:

(1) The variations in the neighborhood geometric properties, such as the curvature and normal vector, are used to find the feature line. For example, Altantsetseg et al. [8] solved the curvature of each point by truncating the Fourier series algorithm. The target feature points were then extracted in combination with a weighted Laplace smoothing algorithm. Demarsin et al. [9] estimated the normal vector of each point using the PCA algorithm, and they segmented the normal vector step points by a region-growing algorithm. Finally, the feature lines were obtained by clustering the step points. Huang et al. [10] estimated the overall directional curvature of the point cloud based on Euler’s theorem. Then, the detection of the edge features was achieved by the directional curvature;

(2) Region segmentation is based on the feature similarity between sampled points and their neighborhood points, and then feature extraction by a contour-extraction algorithm [11]. Wei Zhou et al. [12] used the neighborhood signed-surface-variation (SSV) property to extract feature points. The feature lines were then obtained by connecting the feature points through a minimum spanning tree; Huan Ni et al. [13] implemented the detection of edge points by querying the angular-gap metric between the point and the neighborhood fitting plane. Sampath et al. [14] segmented the building and nonbuilding point clouds into regions, and they then used an improved convex packet algorithm to obtain the feature lines; Bazazian et al. [15] segmented the point clouds based on the geodesic distance, then they defined a multiscale operator based on the local neighborhood properties of the sampled points, and they finally used the operator to determine the feature lines;

(3) Researchers have used surface segmentation by fitting surfaces to obtain feature lines [16]. Kim et al. [17] used the modified MLS method to fit the neighborhood points to obtain the fitted surface. Then, the local principal curvature was estimated by fitting the surface. Finally, the ridge and valley lines were extracted by connecting the ridge vertices along the principal curvature direction, while Lin et al. [18] replaced the neighborhood macro surface by fitting multiple small planes, followed by approximating the point-cloud curvature by fitting multiple small planes, and finally extracting the 3D feature lines based on the curvature mutation. Daniels et al. [19] used the robust MLS method (robust MLS, RMLS) to fit the surface to the neighborhood of each point, calculated the projected residual value of the point on the fitted surface, and finally extracted the feature points by the local maxima of this parameter.

**Figure 1:**
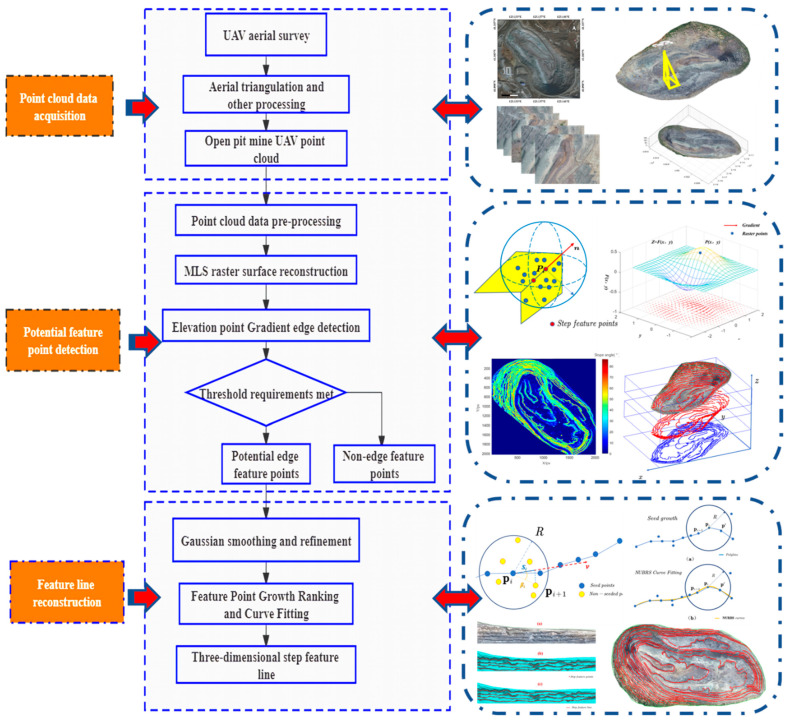
Schematic diagram of the step-feature-line extraction method.

In summary, most edge-feature-extraction methods are applied to mechanical parts [20], urban scenes, and other artificial regular objects. Although the methods are effective at extracting the features of man-made objects, they are susceptible to sampling inhomogeneity, dispersion, and point-cloud-data noise. The influence of these factors can lead to the poor extraction effect of the existing methods for natural terrain field attraction clouds, which are prone to incorrect or missing edge-feature detection, and there is less research on the optimization of the existing detection methods for subsequent feature lines. To this end, an elevation-gradient edge-detection algorithm is introduced to achieve step-feature extraction. The specific steps are as follows: First, the point-cloud data after raster resampling are obtained by preprocessing the open pit, then the elevation gradient is calculated for each elevation point based on the four-direction Sobel operator, and the step-feature points are detected by local adaptive thresholding. Then, the step-feature points are tracked by the seed-growth algorithm to obtain the step-control nodes, and finally, the step-control nodes are fitted by the NURBS algorithm. A schematic diagram of the step-feature-line extraction method is shown in Figure 1.

## 2. Materials and Methods

### 2.1. Open-Pit-Mine Point-Cloud-Data Acquisition

(1) UAV platform

**Figure 2:**
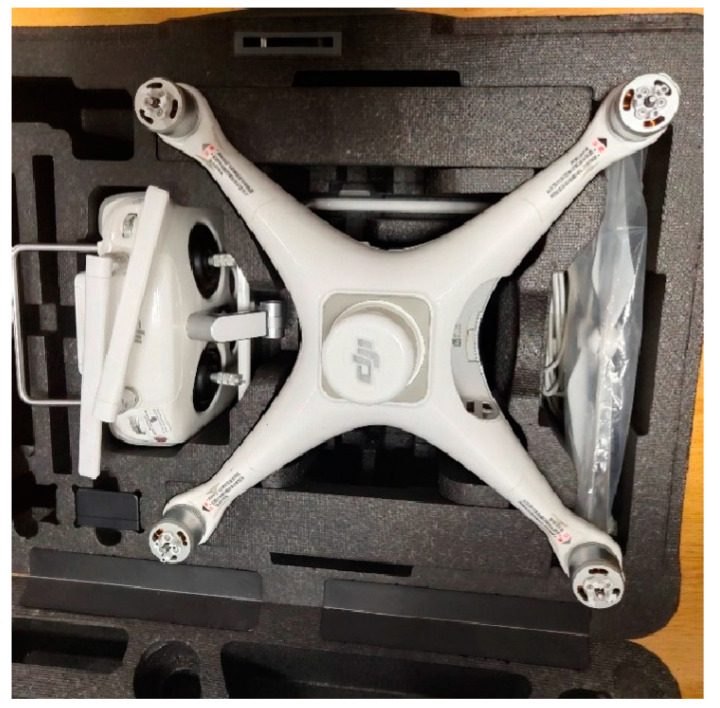
DJI Phantom 4 RTK UAV.

The aerial survey equipment for the research institute consists of a DJI Phantom 4 RTK UAV and the optical image sensor it carries, as shown in Figure 2. The equipment consists of the aircraft, remote control, gimbal camera, and the supporting DJIGS RTK app, which is for low-altitude-photogrammetry applications, with a centimeter-level navigation and positioning system and a high-performance imaging system; its technical parameters are shown in Table 1. The parameters of the graphics workstation used in the data processing are as follows: processor: Intel(R) Core(TM) i9-10900K CPU @ 3.60 GHz; graphics card: NVIDIAGeforce2060; operating system: Windows 10HomeBasic 64-bit; memory: 64 G.

(2) UAV aerial survey

**Figure 3:**
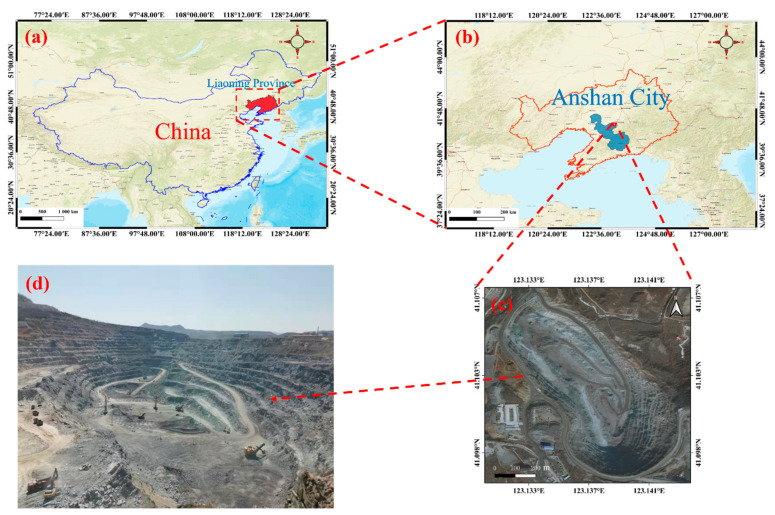
UAV measurement area: (**a**) location of Liaoning Province on the map of China; (**b**) location of Anshan City in Liaoning; (**c**) satellite images of the study area; (**d**) camera images of the study area.

The study area is the Anqian mine Yaba open pit in Anshan city, Liaoning Province, China, and the geographic coordinates of the center of the open-pit mine are 123°08′13″ E and 41°06′11″ N, as shown in Figure 3. The span of the open pit is approximately 1179 m in the north–south direction, and 891 m in the east–west direction, and the maximum mining depth is approximately 212 m. The aerial-survey experimental data were acquired in August 2019, and the flight time zone of the UAV was from 10:00 to 14:00. The flight altitude was 50 m from the elevation of the takeoff point, the ground sampling distance (GSD) was 2.79 cm/pixel, and the flight speed was 6.1 m/s, on average. The heading overlap during image acquisition was set to 70% of the sensor, and the collateral overlap was 70%. The specific aerial-survey parameters are shown in Table 2.

**Figure 4:**
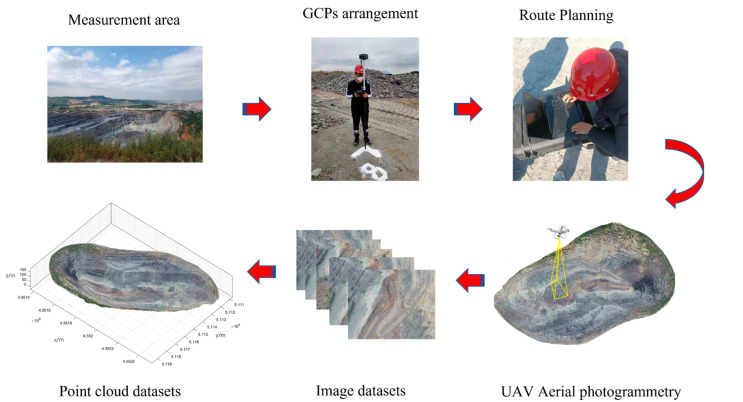
The above six diagrams show the six important steps of the point-cloud-data acquisition process and the sequence, each with a corresponding name for labeling.

In this experiment, first, image control points (GCPs) were laid out for the survey area before the aerial survey (we established thirteen GCPs in different regions, which were used for the geographic alignment of the 3D point cloud to improve the accuracy of the point-cloud data [21]). Then, a tilt-photography aerial survey of the open-pit mines was carried out to obtain the UAV image set. Then, the UAV image set was solved and densely matched by context capture. After the above software operation, the 3D point-cloud data of this experiment were obtained. The specific process of the point-cloud-data acquisition is shown in Figure 4.

### 2.2. MLS Raster Resampling

**Figure 5:**
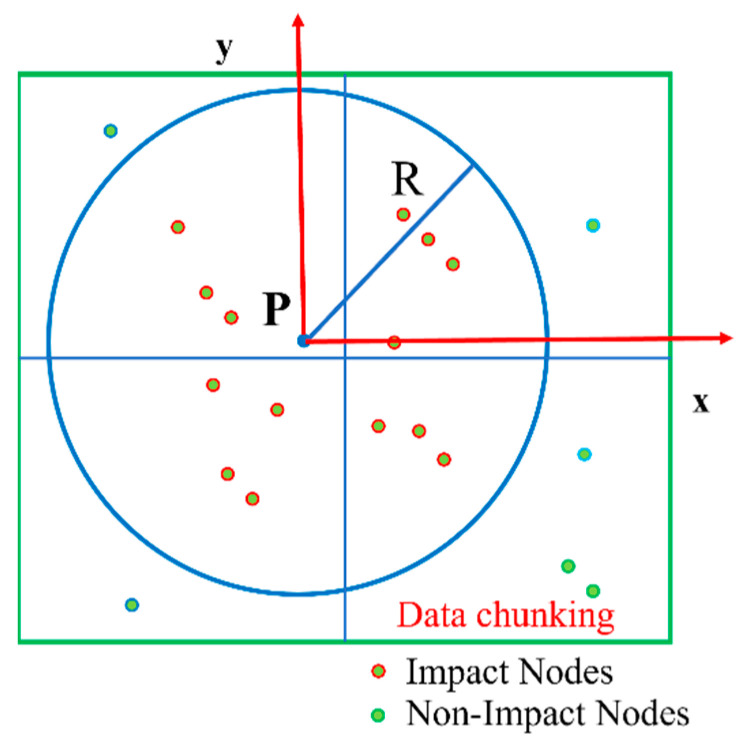
MLS concept map.

Due to the influence of various factors, such as acquisition equipment and environmental conditions, the acquired point-cloud data of the open pit inevitably contain certain noise, and the existence of noise can mask the effective geometric features of the point-cloud data to a certain extent. Due to the discrete and nonuniform sampling nature of the acquired point-cloud data and the complex and variable shape of the open pit, all these factors can have a severe impact on the accuracy and completeness of the step-feature detection. Thus, the MLS (moving least squares) raster resampling of the open-pit point-cloud data is performed prior to the feature extraction to improve the accuracy and completeness of the step-feature detection while reducing noise interference. The method establishes a fit function through the basis function (*p*^T^(*x*)) and coefficient vector (*α*(*x*)) while introducing the concept of tight support (compact support). The value of the fit function at a point is affected only by the nodes in the influence domain at that point, while the nodes outside the influence domain cannot have an impact on it; that is, the nodes outside the influence domain have a weight function of 0. The introduction of the concept of compact support can effectively suppress the influence of noise points on the resampling accuracy. The method dynamically adjusts the magnitude of node weights in the influence domain through the weight function, effectively avoiding the problem of ignoring local features. When the quantity of discrete data is large and the shape is complex, the method does not need to fit and smooth the data in chunks, as other methods do, and it can better characterize the topography of open-pit mines than other methods. The MLS concept diagram is shown in Figure 5.

MLS surface resampling can be described as follows: on the influence domain of the target fit region, the fit function *f*(*x*) is expressed as:

(1) $$f\left(x\right)=\sum_{i=1}^{m}\alpha_{i}\left(x\right)P_{i}\left(x\right)=P^{T}\left(x\right)\alpha \left(x\right)$$

where *P* *∈ U*, with *P*(*x*) = [*P*_1_(*x*), *P*_2_(*x*), …, *P_m_*(*x*)]^T^, where *P*^T^(*x*) is the basis function and is a polynomial of the order *k*; *α*(*x*) = [*α*_1_(*x*), *α*_2_(*x*), …, *α_m_*(*x*)]^T^ is the vector of the coefficients to be found; *m* is the number of terms of the basis function.

Knowing that there are nodes (*X* = [*x*_1_, *x*_2_, …, *x_n_*]^T^) in the influence domain of the target region, the corresponding node values (*Y* = [*y*_1_, *y*_2_, …, *y_n_*]^T^), under which the fitting function (*f*(*x*)) can be solved, are as follows:

(2) $$\begin{array}{c} J|=\sum_{i=1}^{n}w\left(x-x_{i}\right){\left[f\left(x_{i}\right)-y_{i}\right]}^{2}\\ =\sum_{i=1}^{n}w\left(x-x_{i}\right){\left[P^{T}\left(x_{i}\right)\alpha \left(x\right)-y_{i}\right]}^{2} \end{array}$$

where *w*(•) is the weight function of *x*_i_, and *x* − *x*_i_ is the Euclidean distance between nodes. To determine the value of the coefficient (*α*(*x*)), Equation (2) should be taken as a minimal value, and taking the partial derivative of the *α*(*x*) in Equation (2) yields:

(3) $$\frac{\partial J}{\partial \alpha }=A\left(x\right)\alpha \left(x\right)-B\left(x\right)y=0$$

(4) $$\alpha \left(x\right)=A^{-1}\left(x\right)B\left(x\right)y$$

where

(5) $$A\left(x\right)=\sum_{I=1}^{n}w\left(x-x_{I}\right)p\left(x_{I}\right)p^{T}\left(x_{I}\right)$$

(6) $$B\left(x\right)=[w\left(x-x_{1}\right)p\left(x_{1}\right),w\left(x-x_{2}\right)p\left(x_{2}\right),\ldots ,w\left(x-x_{n}\right)p\left(x_{n}\right)]$$

(7) $$y^{T}=\left[y_{1},y_{2},\ldots ,y_{n}\right]$$

By substituting Equation (4) into Equation (1), the fitted function (*f*(*x*)) can be obtained.

The weight function decreases with increasing distance. The weight function is a compact support. The weight function is zero outside the domain of the influence of *x_i_*, and the weights need to be monotonically decreasing as ||*x* − *x_i_*||^2^ increases; for this reason, a Gaussian weight function is chosen to resample the data, which can be expressed as:

(8) $$w_{i}\left(x\right)=\frac{e^{-ud_{i}^{2}\left(x\right)}}{d_{i}^{2}\left(x\right)}$$

where *d_i_(x)* denotes the distance between the *i*-th original sampling point (*x_i_*) and the resampling point (*x*), and *u* denotes the influence factor between the features in the influence region and the resampling.

When rasterizing the point-cloud data, it is also necessary to set the range and step size of the raster. The range of the raster can be defined according to the size of the outer rectangle of the open-pit mine, which is determined by the maximum value of the *x* and *y* coordinates of the open-pit-mine point cloud (*x*_max_, *y*_max_) (*x*_min_, *y*_min_), and the step length of the raster is determined according to the horizontal average distance of the open-pit-mine point cloud.

### 2.3. Feature-Point Detection

**Figure 6:**
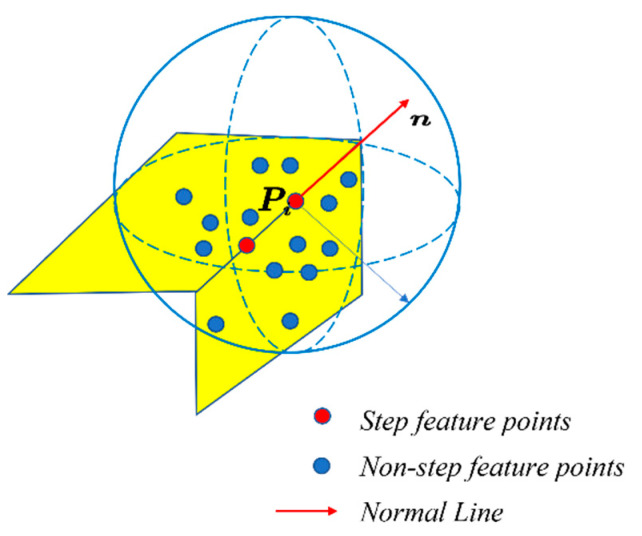
Step-feature-point detection.

Feature-point detection is a key step in extracting step-feature lines. In this study, a step-feature-line extraction method based on elevation-gradient edge detection is proposed, which is referred to as EGED-CS below. This extraction method detects the step-feature points by the neighborhood-elevation-gradient change. The gradient of a point is usually defined as the direction in which the scalar field of the point grows fastest, and the magnitude of the gradient is taken as the maximum rate of change in the scalar value in that direction. For an open-pit-mine point cloud, the greater the change in the elevation gradient at a point, the more pronounced the edge feature at that point. For ease of description, points where the neighborhood elevation gradient varies above a specific threshold are defined as abrupt change points. Moreover, step edges are usually found where there is a high density of abrupt change points. Therefore, step-feature points can be detected by the neighborhood-elevation-gradient mutation feature. The step-point detection is shown in Figure 6.

The research method draws on the ideas of region segmentation and edge detection in image processing, and it is derived from two-dimensional detection to three-dimensional detection. The step-feature points are extracted by performing gradient detection on the resampled elevation information. The edge-detection technique is the most fundamental technique in fields such as image processing and computer vision. The edge-detection algorithm uses the law that the first-order derivative at the edge takes the extreme value, and the second-order derivative takes the extreme value at the step-like edge to detect the edge. The algorithm is implemented by constructing the edge-detection operator for some small fields of pixels in the original image. The commonly used edge-detection operators are the Roberts operator, Sobel operator, Prewitt operator, Laplacian operator, and LOG operator. One of the most classic Canny algorithms is the multilevel edge-detection algorithm developed by John F. Canny in 1986 that created the computational theory of edge detection [22]. Most of the subsequent edge-detection algorithms have been continuously updated and improved based on the ideas of Canny’s algorithm. Currently, edge-detection techniques are widely used in many fields. These include edge-feature extraction for medical images, license-plate recognition, and face-detection techniques [23,24].

To accurately detect the step-feature points, the point-cloud data after raster resampling can be set as a two-dimensional discrete function: *Z* = *F*(*x*, *y*). Then, for any point (*P*(*x*, *y*) ∈ *Z*), a corresponding elevation gradient can be calculated as:

(9) $$\nabla F\left(x,y\right)={\left[d_{x},d_{y}\right]}^{T}={\left[\frac{\partial F}{\partial x},\frac{\partial F}{\partial y}\right]}^{T}$$

where *d_x_* and *d_y_* are the first-order partial derivatives of *F(x*, *y)* along the horizontal and vertical directions, respectively.

The magnitude of the elevation gradient at a point is the norm of the corresponding partial derivative at that point in the direction of the maximum rate of change, and it can be expressed as:

(10) $$mag\left(\nabla F\right)=grad\left(x,y\right)=\sqrt{\frac{\partial^{2}F}{\partial x^{2}}+\frac{\partial^{2}F}{\partial y^{2}}}$$

Because *F*(*x*, *y*) is a discrete two-dimensional function and the smallest differentiating cell is the raster step, the following formula can be used to approximate the differentiation of *F*(*x*, *y*) for this purpose:

(11) $$\begin{array}{c} \frac{\partial F\left(x,y\right)}{\partial x}=F\left(x+1,y\right)-F\left(x,y\right)=d_{x}\\ \frac{\partial F\left(x,y\right)}{\partial y}=F\left(x,y+1\right)-F\left(x,y\right)=d_{y}| \end{array}\;$$

The above approximate-differentiation operation can be achieved by detecting the operator template for the *F*(*x*, *y*) convolution calculation. However, the Sobel operator in the traditional algorithm is not sensitive to edge features in other directions because it only has templates for horizontal and vertical directions. This results in poor feature-extraction results for complex terrain and variable tilt angles in the edge space. To address the above problems, this study adds two-directional templates of 45° and 135° to the original template, forming a four-directional Sobel-operator template. The templates for the four directions are shown as follows:

(12) $$f_{x}\left(x,y\right)=\left[\begin{array}{c} \begin{array}{ccc} -1 & 0 & +1\\ -2 & 0 & +2\\ -1 & 0 & +1 \end{array} \end{array}\right],f_{y}\left(x,\;y\right)=\left[\begin{array}{ccc} -1 & -2 & -1\\ 0 & 0 & 0\\ +1 & +2 & +1 \end{array}\right]$$

(13) $$f_{45{}^{\circ}}\left(x,y\right)=\left[\begin{array}{c} \begin{array}{ccc} -2 & -1 & +0\\ -1 & 0 & +1\\ 0 & +1 & +2 \end{array} \end{array}\right],f_{135{}^{\circ}}\left(x,\;y\right)=\left[\begin{array}{ccc} 0 & -1 & -2\\ +1 & 0 & -1\\ +2 & +1 & 0 \end{array}\right]$$

**Figure 7:**
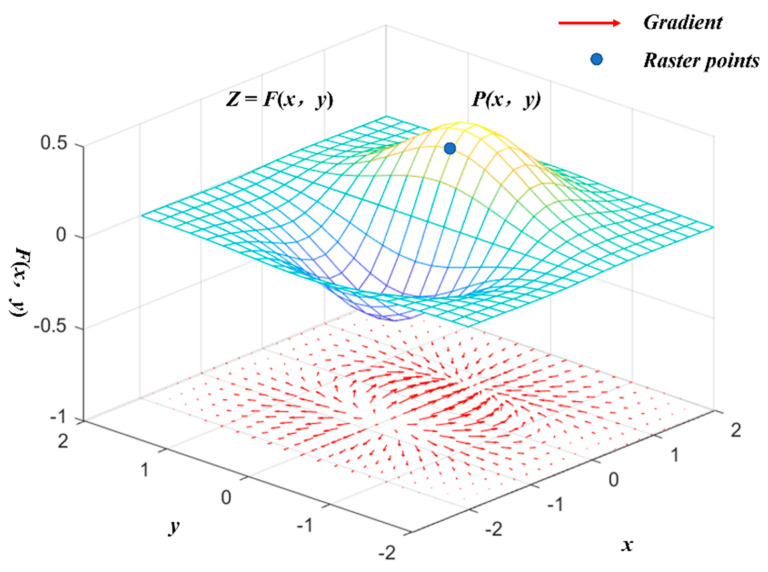
Gradient calculation.

The four-directional Sobel-operator template is used to traverse the raster point cloud from left to right and from top to bottom, and to perform a convolution operation to obtain the elevation-gradient magnitude at each point. The amplitude-calculation formula of the elevation gradient is as follows:

(14) $$d_{n}=F\left(x,\;y\right)*f_{n}\left(x,\;y\right)$$

(15) $$edge\left(x,\;y\right)=\sum_{n=1}^{4}\left|d_{n}\right|$$

where *f_n_*(*x*, *y*) represents the operator template in four different directions, and *edge*(*x*, *y*) is the magnitude of the elevation gradient at that elevation point. The elevation-gradient calculation is shown in Figure 7.

Canny detection methods usually set global thresholds based on prior knowledge. When extracting step size features from point-cloud data with complex features, the Canny detection method easily leads to incomplete step-size-feature detection or the introduction of nonstep-size-feature points. To address this shortcoming, this study proposes a local adaptive dynamic threshold determination method. The optimal elevation-gradient-detection threshold for the location is determined based on the distribution of the elevation-gradient information in the surrounding neighborhood. The adaptive thresholds are calculated as follows:

(16) $$T=\frac{\left[sum\left(A\right)-min\left(A\right)-max\left(A\right)\right]}{7}\;$$

where *A* represents the 3 × 3 template matrix of elevation-gradient magnitudes, *sum*(*A*) is the sum of the elevation-gradient magnitudes of all points in the template, *min*(*A*) is the minimum value of the elevation-gradient magnitude in the template, and *max*(*A*) is the maximum value of the elevation-gradient magnitude in the template.

As there are some pseudostep terrains with small surface undulations or small slopes in the open-pit mines, the spatial elevation gradient variation characteristics of these small slopes are more similar to those of steps. For this reason, there are some pseudostep feature points in the above detection results. Considering that the slopes of these pseudostep terrains are small, while the slopes of step terrains are usually steep, the detection results of the step-feature points are optimized by setting a slope threshold to filter out nonstep-edge-feature points to improve the detection accuracy. The slope angle is generally calculated using the fitted surface method, where the slope matrix is obtained by fitting a quadratic surface to the eight neighborhoods of each elevation point, and the slope is calculated as follows:

(17) $$Slope=tan\sqrt{Slope_{x}{}^{2}+Slope_{y}{}^{2}}$$

(18) $$\theta =\frac{Slope}{\pi }\times 180{}^{\circ}$$

where *θ* is the slope angle, *Slope* is the slope, *Slope_x_* is the slope in the *x*-direction, and *Slope_y_* is the slope in the *y*-direction.

### 2.4. Step-Feature-Line Reconstruction

The step-feature line is an ordered connection of the step-feature points according to the actual direction of the step. Because the extracted step-feature points are scattered and disordered and do not contain spatial topological relationships, it is necessary to trace some valid step-feature points that represent the step-feature-line orientation by certain methods. We call these effective step-feature points step-control nodes. Finally, the step-control nodes are ordered and fitted to reconstruct the step-feature lines.

(1) Step-control-node tracking

**Figure 8:**
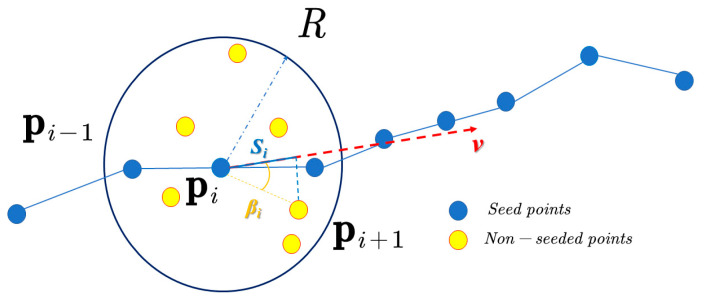
Seed-growth tracking.

To accurately reconstruct the step-feature lines, a seed-growth-based step-control-node tracking method [25,26] is introduced, as shown in Figure 8. The method is based on the seed-growth algorithm to track out the step-control nodes in the step-feature-point set (O), and to rank the step-control nodes according to the spatial orientation. The step-control-node tracking method is implemented in the following steps.

(1) First, a spherical-local-influence region with the radius size (*R*) is constructed, and then the seed-point tracking is completed by passing the spherical-local-influence region. The radius (*R*) depends on the average distance (*d_min_*) of the point cloud, which is calculated as follows:

(19) $$d_{min}=\frac{\sum_{i=1}^{N}\;d\left(p_{i}\;,q_{i}^{\prime }\right)}{N}$$

(20) $$R=n\cdot d_{min}$$

where *N* is the total number of points, *q_i_*_′_ is the nearest point of *p_i_*, *d*(*p_i_*, *q_i′_*) is the distance between points *p_i_* and *q_i_*_′_, and *n* is the sphere-influence factor, which controls the sphere-influence range;

(2) We initialize the step-control-node queue (*C*), select the point (*p*_0_) from the feature point set (*O*) as the initial seed point, and join the queue (*C*). Then, we construct a spherical-local-influence region of the radius (*R*) with the seed point (*p*_0_) as the center point, track the set of nearest-neighbor points (*OR*(*q*) = {*q_i_*| *q_i_* ∈*O*, | *q_i_* − *p*_0_| < *R*}) within this region, and then perform PCA principal element analysis [27] on the *OR* to obtain the principal axis vector (*v*), and to calculate the projection angle (*β_i_*) and the projection distance (*S_i_*) for each point (*q_i_*) in the *OR* in the positive direction of the principal axes, while setting the angle threshold (*θ*). If *β_i_* < *θ* and *S_i_* = *max* {*S_1_*, *S*_2_, …, *S_n_*}, then *q_i_* is selected as the nascent seed point, and *q_i_* is added to the queue (*C*);

(3) Then, the newborn seed point (*q_i_*) is taken as the current object, and the tracking is continued by repeating step (2). The above steps are cycled until the newborn seed point cannot be tracked in the positive direction, and the tracking starts from the original seed point (*p*_0_) in the opposite direction. When *β_i_* > *θ*, the current direction tracking is stopped, the current point is recognized as the branch-line feature point, and the tracking is completed in the branch line.

(2) Step-feature-line fitting

A number of step-control-node queues (*C_k_*) can be obtained by the above tracing steps. If these step-control nodes are directly connected, the generated step-feature lines cannot achieve the expected accuracy and effect, and so the step-feature lines need to be smoothed and optimized. In practical engineering applications, the nonuniform rational B-spline (NURBS) algorithm can accurately describe the pose of a spatial curve [28,29,30], and so this study introduces the NURBS algorithm to fit and optimize the step-feature lines. The mathematical definition of a NURBS curve is shown below:

(21) $$P\left(u\right)=\frac{\sum_{i=1}^{n}\omega_{i}d_{i}N_{i,k}\left(u\right)}{\sum_{i=1}^{n}\omega_{i}N_{i,k}\left(u\right)},0\leq u\leq 1$$

where *P(u)* is the position vector on the curve, *n* is the number of control nodes, ω*_i_*(*i* = 0, 1, …, *n*) is the weight factor, *d_i_*(*i* = 0, 1, …, *n*) is the control node, and *N_i_*, *_k_(u)* is the *k*th B-spline basis function controlled by the node vector.

Let the set of step-feature-line feature points obtained after the above seed-tracking sorting process be *C* = {*q*_0_, *q*_1_, …, *q_i_*, …, *q_m_*|*i* < *m*, *q_i_* ∈ *C*}; substituting this point set into the above equation yields:

(22) $$\left[\begin{array}{ccc} N_{0,k}\left(u_{0}\right) & \cdots & N_{n,k}\left(u_{0}\right)\\ N_{0,k}\left(u_{1}\right) & \cdots & N_{n,k}\left(u_{1}\right)\\ \vdots & \ddots & \vdots \\ N_{0,k}\left(u_{m}\right) & \cdots & N_{n,k}\left(u_{m}\right) \end{array}\right]\left[\begin{array}{c} d_{0}\\ d_{1}\\ \vdots \\ d_{n} \end{array}\right]=\left[\begin{array}{c} \omega_{0}q_{0}\\ \omega_{1}q_{1}\\ \vdots \\ \omega_{m}q_{m} \end{array}\right]$$

As the weight factor (*ω_i_*) is usually 1 in practical applications, Equation (22) can be changed to *Nd = q*. At this point, the curve-control vertex (*d*) can be expressed as:

(23) $$d={\left(N^{T}N\right)}^{-1}N^{T}q\;$$

**Figure 9:**
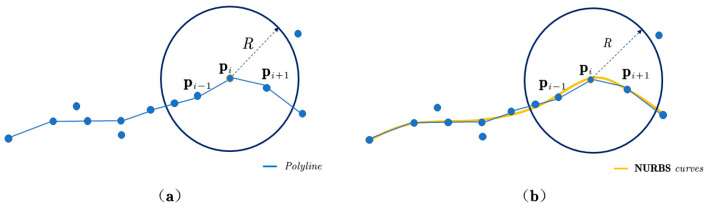
(**a**) The blue line is a step-feature line without smoothing, (**b**) the yellow curve is a step-feature line optimized by NURBS.

Once the control vertex (*d*) of the NURBS curve is determined, the fitted step-feature curve is obtained, as shown in Figure 9.

### 2.5. Step-Feature-Line Extraction-Result-Verification Method

**Figure 10:**
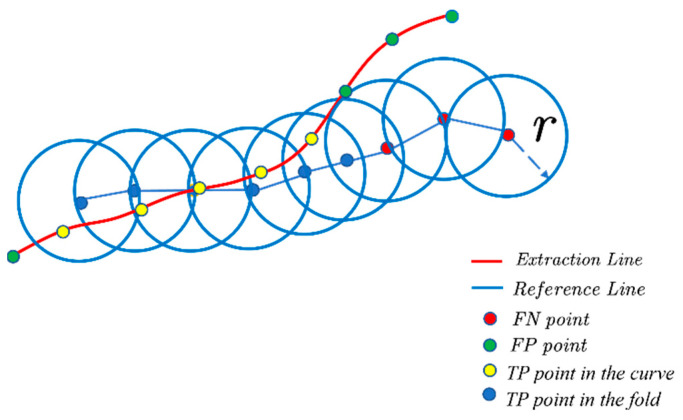
Line-feature-accuracy comparison method.

To evaluate the extraction quality of the step-feature lines, the evaluation methods in the literature [12,31,32,33,34] were referred to for the quantitative assessment of the step-feature-line extraction results. The step-feature lines obtained by visual interpretation were used as the reference line. As shown in Figure 10, the evaluation method obtains equally spaced points on the reference line and extraction line, creates a spherical buffer of the radius size (*r*) for the equally spaced points on the reference line, matches the equally spaced points on the extraction line with the minimum-distance mapping, and determines the extraction accuracy by three indicators: the completeness rate (*α*), accuracy rate (*β*), and overall quality (*γ*):

(24) $$\alpha =\frac{TP}{TP+FN}\;$$

(25) $$\beta =\frac{TP}{TP+FP}\;$$

(26) $$\gamma =\frac{TP}{TP+FN+FP}$$

where *TP* is the number of correctly classified points in the step point set, *FN* is the number of incorrectly classified points in the step point set, and *FP* is the number of incorrectly classified points in the nonstep point set.

## 3. Results

### 3.1. UAV Point-Cloud Data

**Figure 11:**
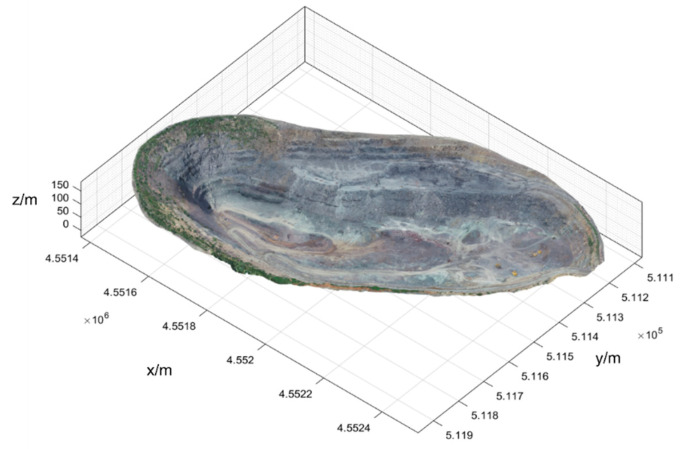
Point-cloud model of open-pit mines.

The UAV aerial-survey experiment can obtain the UAV image set of the survey area, and after context capture to carry out the null-three decomposition and dense matching on this image set, the 3D point-cloud data of this experiment can be obtained. The point-cloud data of the nonopen-pit area are cropped, and only the point-cloud data of the open pit are retained, which can form the open-pit point-cloud model shown in Figure 11. The relevant parameters of this point-cloud dataset are shown in Table 3.

### 3.2. Data Preprocessing

**Figure 12:**
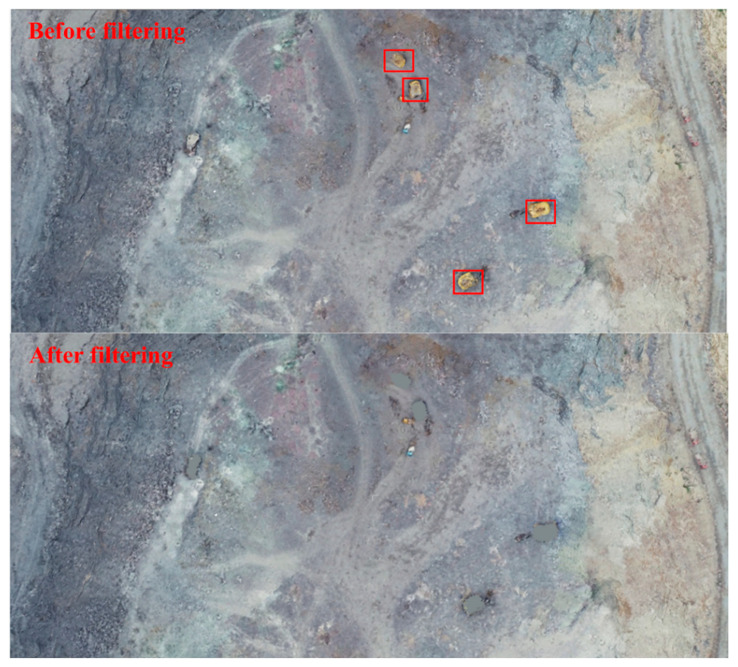
Comparison of results of nonsurface target rejection in the open-pit mine (large-mine-car point clouds in red boxes are filtered out).

To exclude the influence of nonsurface targets on the detection accuracy of step features, such as large mining cars in the open pit, the nonsurface targets are rejected by referring to the mining-car-extraction method in the literature [35]. First, according to the actual terrain of the open-pit point cloud and the size of the nonground targets to be extracted, the slope (s) is set to 1, the minimum height-difference threshold (*h_min_*) is 0.5 m, the maximum height-difference threshold (*h_max_*) is 3 m, and the maximum window size is 8 m; then, the nonground targets in the open-pit-mine point cloud are extracted and rejected by progressive morphological filtering. Finally, the mean elevation of the neighboring points of the rejected targets is used to replace the elevation values of the rejected points, thus filling the rejected point sets and forming new open-pit-mine point-cloud data. A comparison of before and after the rejection of nonsurface targets in the open-pit mines is shown in Figure 12.

**Figure 13:**
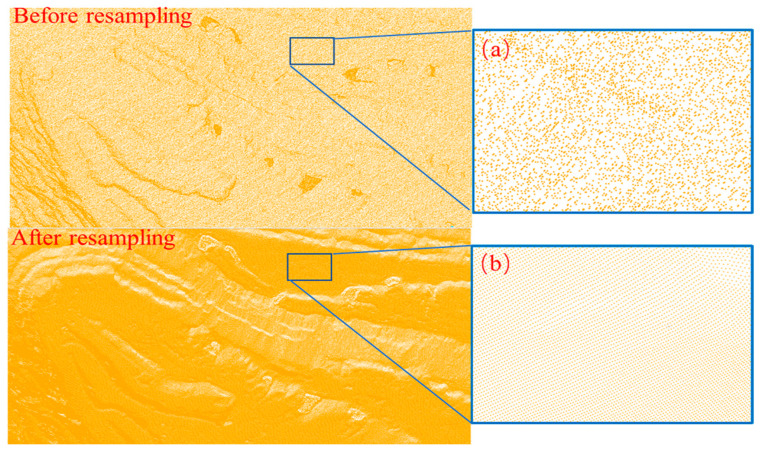
Comparison of MLS resampling results: (**a**) point-cloud details before resampling, (**b**) point-cloud details after resampling.

After the nonground targets have been removed and filled in, the new open-pit-mine point-cloud data can be resampled in a raster based on the MLS algorithm. First, the resampling raster step was set to 0.5 m, and the raster range was {*x*, *y*|511,070.2 < *x* < 511,961.4, 4,551,369.6 < *y* < 4,552,548.8}. The raster points were then searched in the original point cloud based on a *k-d* tree with a nearest-neighbor search radius of 2 m. Finally, the results of the neighbor search were resampled using a quadratic basis: *P* = [1, *x*, *y*, *x^2^*, *xy*, *y^2^*]^T^, as moving squares. The MLS resampling results are shown in Figure 13.

### 3.3. Step-Feature-Point Detection

**Figure 14:**
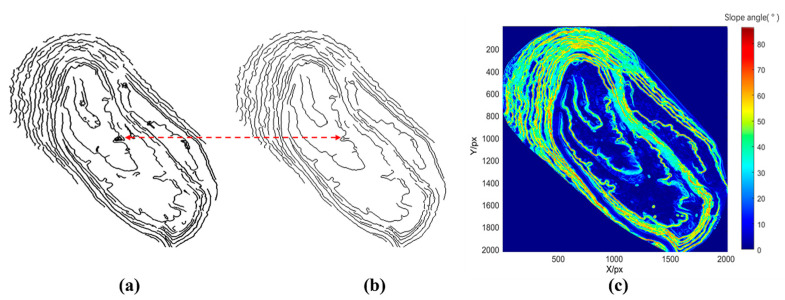
(**a**) Detection results without slope-threshold optimization; (**b**) detection results of step-feature points with slope-threshold optimization; (**c**) slope distribution of the open-pit mine.

Step-feature-point detection is achieved by using the four-directional Sobel operator to calculate the elevation gradient at each point by local dynamic thresholding. First, the operator is used to convolve the open-pit raster point cloud to obtain the elevation-gradient magnitude (*edge(x*, *y)*) for each raster point (*P(x, y)*). Next, raster points with the *edge(x, y)* above a local adaptive threshold (T) are labeled as step-feature points, thus obtaining a two-dimensional distribution of step-feature points, as shown in Figure 14a.

As there are some small-scale ground undulations or small slopes of pseudostep terrain in the open-pit mines, the spatial elevation gradient variation characteristics are more similar to those of steps. The clumped flocculent lines indicated by the red arrows in Figure 14a are the interference lines of the pseudostep terrain on the step-feature detection, which are the interference lines of pseudostep terrain on step-feature detection. If these lines are not removed in time, then they will have a great impact on the subsequent step-line-extraction accuracy. Because the slopes of these pseudostep terrains are generally small, they can be removed by setting the slope threshold to obtain more realistic step-feature points. The slope matrix is obtained by fitting a quadratic surface to the eight neighborhoods of each raster point on *F(x, y)*, and finally, the slope angle corresponding to each raster point can be calculated according to the slope matrix by Equations (17) and (18). The slope distribution of an open-pit mine can be drawn according to the magnitude of the slope angle, as shown in Figure 14c. To ensure the stability of the slope, the safety range of the step-slope angle is usually set within 75° in the mining industry. The threshold value of the step face, road and flat-face-slope division is usually 30°. Therefore, the slope-angle threshold is set to 30°–75°, and the feature points with slope-angle sizes outside this range are eliminated to extract the step-feature points that meet the slope-angle requirement. The results of the step-feature-point detection after slope-threshold optimization are shown in Figure 14b.

**Figure 15:**
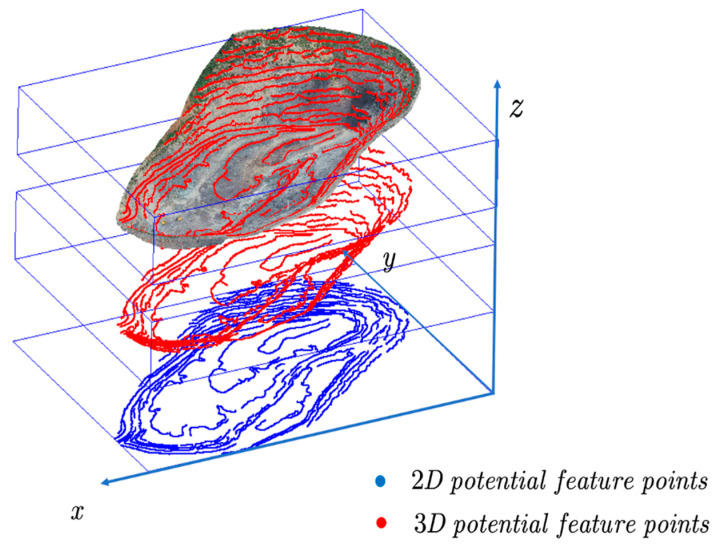
Step-feature-point conversion (the lower blue part is the extracted 2D step-feature points, the middle red part is the 2D step-feature points converted to 3D form, and the upper part is the 3D step-feature points overlaid with the open-pit point cloud).

The step-feature points after the above slope-angle-optimization process are two-dimensional data, while the step-feature points should be three-dimensional spatial data. For this reason, the index (*x*, *y*) of the plane coordinates corresponding to the step-feature point (*P_I_*) is searched first, then the corresponding elevation (*h*) in the raster point cloud is queried according to this index, and its three-dimensional coordinates (*x*, *y*, *h*) are recorded. By repeating the above iterative query operation for each step-feature point (*P*), the final three-dimensional step-feature-point set (*O*) can be obtained, as shown in Figure 15.

### 3.4. Step-Feature-Line Reconstruction

**Figure 16:**
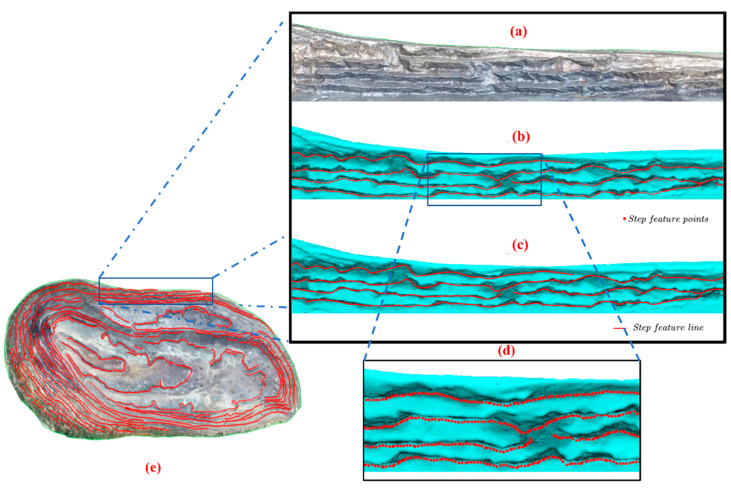
Step-feature-line reconstruction process: (**a**) part of the real model of the open-pit mine; (**b**) red points are the extracted step-feature points; (**c**) red line is the fitted step-feature line; (**d**) a zoomed-in view of the area, for the clear visibility of the step-feature points; (**e**) all the step-feature lines of the experimental area.

The step-feature points can be obtained by the above steps. The actual step-feature line can be obtained only by orderly connecting the step-feature points according to the actual direction of the step. The study uses a seed-growth algorithm for the control-node tracking of the step-feature-point set (*O*). In this process, first, the average distance (*d_min_*) of the open-pit-mine point cloud was calculated to 0.4063 m. Then, the tracking influence coefficient (*n*) was set to 5, and the maximum number of tracking nearest-neighbor points (*k*) was 10. Then, the spherical influence radius (*R*) was 2.0315 m. To avoid the overly large and undersized angle threshold (*θ*) that leads to too smooth and too linear fitting results, the *θ* was set in the range of 20°–30° to improve the step-feature-line fitting effect. Based on the above parameters, the seed-tracing algorithm was used to obtain the control nodes and node sequences of the step-feature line. Finally, the NURBS algorithm was used to smoothly fit the control nodes to obtain a smooth step-feature line, and the step-feature-line reconstruction process is shown in Figure 16.

## 4. Discussion

### 4.1. Visual Interpretation Accuracy Evaluation

**Figure 17:**
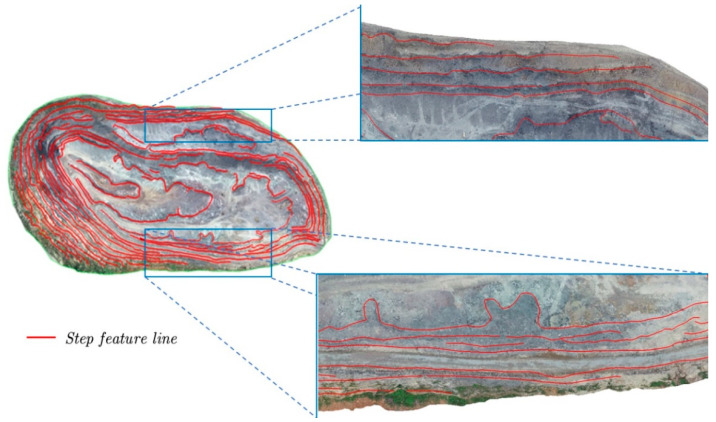
Step-feature lines (the general overhead view has been bolded for clarity).

As shown in Figure 17, the red lines are the step-feature lines extracted by the EGED-CS method, and the step-feature-line overlay is displayed on a texture-mapped irregular triangular network (TIN). Visual inspection shows that the EGED-CS method can extract most of the step-feature lines.

**Figure 18:**
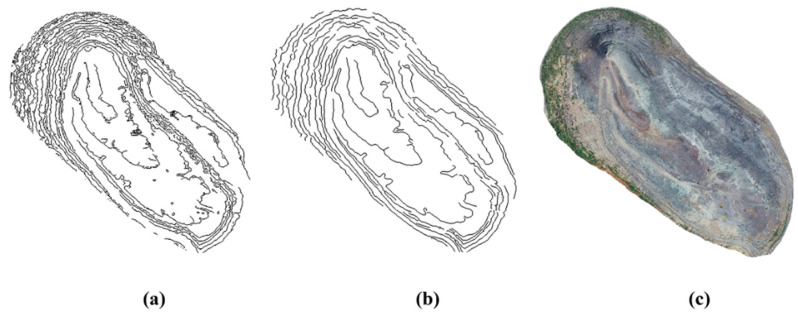
(**a**) Detection result of Canny algorithm; (**b**) detection result of EDEG-CS method; (**c**) real view of point cloud in the open-pit mine.

To verify the effectiveness of the EGED-CS method on the step-edge extraction, it was compared with the Canny algorithm. Figure 18 shows that the EGED-CS method does a better job of avoiding noise interference and retaining more edge information. The Canny algorithm is sensitive to noise, which leads to many nonreal step edges in the step-detection results, while the EGED-CS method can avoid noise interference and accurately obtain more realistic step-edge information. Additionally, the EGED-CS method detects more edge pixels and has a better effect on the edge pixel connections. Experiments show that this method is more effective and adaptive than the Canny algorithm for extracting step edges from open-pit point clouds.

**Figure 19:**
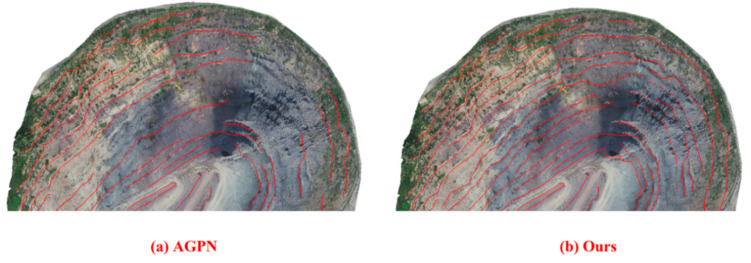
Step-feature lines in the cliff area: (**a**) extraction result of AGPN method; (**b**) extraction result of EDEG-CS method.

To verify the effectiveness of the EGED-CS method in terms of feature-line extraction, it was compared with the analyzing geometric properties of neighborhoods (AGPN) method proposed in the literature [36] (the seed-growth algorithm cannot be implemented effectively due to the high number of noisy step features extracted by the Canny algorithm and the high number of interfering factors, which lead to the inability to automatically generate step-feature lines, and so the evaluation of the Canny algorithm regarding step-feature lines cannot be performed). The comparison results show that the difference between the results of the step-feature lines extracted by the two methods is not obvious in the region where the step features are obvious. For visualization, an example of the area where the difference between the extraction results of the two methods is more obvious was selected, as shown in Figure 19 below. The extraction results obtained by the AGPN method (shown in Figure 19a) are compared with those obtained by the EGED-CS (Figure 19b); in the steep region, the AGPN algorithm cannot completely extract some of the step-feature lines, while the research method can completely and effectively extract this part of the step-feature lines. EGED-CS is significantly better than the AGPN method in the steep region.

### 4.2. Quantitative Accuracy Evaluation

To quantitatively evaluate the extraction effect of step-feature lines, the completeness (*α*), accuracy (*β*), and overall quality (*γ*) are calculated for the EGED-CS method and AGPN, and the extraction effect is judged by these three indexes. In practice, the three evaluation indicators depend on the size of the spherical buffer radius (*r*). The choice of the buffer-radius size in reference [12] is studied, the buffer radius (*r*) is set to 1.5 *d_min_*, and the distance between equally spaced points is 3 *d_min_*, according to the actual situation.

As seen in Table 4, the extraction completeness (α) of the AGPN method is 80.11%, the accuracy (*β*) is 87.23%, and the overall quality (*γ*) of the extraction is 71.70%. In contrast, the EGED-CS method has an extraction completeness rate (α) of 90.34%, an accuracy rate (*β*) of 91.25%, and an overall quality of extraction (*γ*) up to 83.14%. The evaluation results show that the EGED-CS method can extract the open-pit step-feature lines completely and accurately, and it is better than the AGPN method in terms of completeness, accuracy, and overall quality. The AGPN method is significantly lower in completeness and overall quality. This is mainly because the method emphasizes combining the geometric properties of the query-point neighborhood with the angular-gap metric of the fitting plane to achieve edge detection. Its step-feature-detection accuracy mainly depends on the accuracy of the plane fitting, which is more sensitive to the size of the neighborhood range. This leads to the requirement of different values of the neighborhood range for the detection of different types of step features, and so it is difficult for the AGPN method to completely extract all types of step features for such complex scenes as open-pit mining. This study uses MLS for the resampling optimization of open-pit mines to avoid the effects of sampling inhomogeneity, dispersion, and noise on the detection accuracy of the step features. Additionally, different types of step features are detected according to local adaptive thresholds. This adaptive detection can effectively improve the detection completeness of various complex step features, thus compensating for the defect of the incomplete step-feature lines detected by the AGPN method.

In summary, the EGED-CS method has a greater improvement in the step-edge-detection effect than the Canny algorithm. The method also has obvious advantages over the AGPN algorithm in the step-feature-line extraction effect. In addition, it can automatically extract the step-feature lines of the open-pit mine completely and accurately.

## 5. Conclusions

In this study, we analyzed the key problems of low accuracy, local-feature-line loss, and the discontinuity of open-pit step-feature-line extraction. An automatic extraction method of open-pit step-feature lines based on neighborhood-elevation-gradient analysis was proposed. A comparative analysis with the Canny and AGPN methods was carried out. Based on the analysis from the perspective of visual interpretation, the EGED-CS-method step-feature-line extraction effect was significantly better than the Canny algorithm and AGPN algorithm in the two-dimensional plane results, and the extraction accuracy was higher. Based on the analysis from the perspective of quantitative evaluation, the extraction completeness (*α*) of the AGPN method was 80.11%, the accuracy (*β*) was 87.23%, and the overall quality of extraction (*γ*) was 71.70%, while the extraction completeness (*α*) of the EGED-CS method was 90.34%, the accuracy (*β*) was 91.25%, and the overall quality of extraction (*γ*) reached 83.14%, with significantly better accuracy than the former. The research results show that the EGED-CS method can automatically extract the surface open-pit-mine step-feature lines completely and accurately, which provides important technical support to realize the automatic drawing of the open-pit-mine status map of the surface open-pit mine.

## Figures and Tables

**Table 1:** Technical parameters of UAV and optical sensors.

Aerial-Survey Equipment	Parameter	Value
UAV	Maximum takeoff weight	1391 g
	Flight time	Approximately 30 min
	Hovering accuracy	Vertical: ±0.1 m; horizontal: ±0.1 m
	Maximum horizontal flight speed	50 km/h (positioning mode), 58 km/h (attitude mode)
	Satellite positioning module	GPS+BeiDou+Galileo (Asia region)
		GPS+GLONASS+Galileo (other areas)
	Maximum operating area in a single flight	Approximately 1 km^2^
	Maximum flight altitude	600 m
Camera	Pixel	20 million effective pixels (20.48 million total pixels)
	Image sensor	1-inch CMOS
	Lens	FOV: 84°, 8.8 mm/24 mm
		(35 mm-format equivalent)
		Aperture f/2.8–f/11 with autofocus
		(focus distance: 1 m–∞)
	Maximum photo resolution	5472 × 3078 (16:9)
		4864 × 3648 (4:3)
		5472 × 3648 (3:2)
	Image format	JPEG

**Table 2:** UAV aerial-survey parameters.

Aerial-Survey Parameters	Value
Heading-overlap rate	70%
Bypass-overlap rate	70%
Image resolution	4864 × 3648
Ground sampling distance (GSD)	2.79 cm/pixel
Tilt angle Flight speed (average)	−45° 6.1 m/s
Flight altitude (average)	50 m

**Table 3:** Parameters related to the point-cloud dataset.

Parameters	Values
Number of point clouds	1,532,489
Point-cloud density (pts/m^2^)	1.92
Average point distance (m)	0.4063
Scope (m × m)	891 × 1179
Elevation (m)	−25–186

**Table 4:** Comparison of step-edge-detection accuracy results.

Method	*TP*	*FP*	*FN*	*α*	*β*	*γ*
EGED-CS	19,097	1832	2042	90.34%	91.25%	83.14%
AGPN	16,934	2497	4205	80.11%	87.23%	71.70%

## Data Availability

The data presented in this study are available upon request from the corresponding author. The data are not publicly available due to privacy issues.
